# Supplementary material for: Resolving the full spectrum of human genome variation using Linked-Reads
Source: Genome Res. 2019 Apr;29(4):635–45. doi: 10.1101/gr.234443.118 (PMC6442396; doi:10.1101/gr.234443.118)
Supplement: Supplemental Material [file supp_gr.234443.118_Supplemental_longranger-2.2.zip › longranger-2.2.tar.bz2!/lr2.2-public/lib/stan/cmdstan/stan/doc/api/html/init__adapt_8hpp_source.html]

Stan: src/stan/common/init\_adapt.hpp Source File


|  |  |
| --- | --- |
|  | Stan  2.5.0  probability, sampling & optimization |


- Main Page
- Related Pages
- Namespaces
- Classes
- Files

- File List
- File Members

All Classes Namespaces Files Functions Variables Typedefs Enumerations Enumerator Friends Macros Pages

- src
- stan
- common

init\_adapt.hpp

Go to the documentation of this file.

1 #ifndef STAN\_\_COMMON\_\_INIT\_ADAPT\_HPP

2 #define STAN\_\_COMMON\_\_INIT\_ADAPT\_HPP

3

4 #include <stan/math/matrix/Eigen.hpp>

5 #include <stan/mcmc/base\_mcmc.hpp>

6 #include <stan/gm/arguments/categorical\_argument.hpp>

7 #include <stan/gm/arguments/singleton\_argument.hpp>

8

9 namespace stan {

10  namespace common {

11

12  template<class Sampler>

13  bool init\_adapt(Sampler\* sampler,

14  const double delta,

15  const double gamma,

16  const double kappa,

17  const double t0,

18  const Eigen::VectorXd& cont\_params) {

19  const double epsilon = sampler->get\_nominal\_stepsize();

20

21  sampler->get\_stepsize\_adaptation().set\_mu(log(10 \* epsilon));

22  sampler->get\_stepsize\_adaptation().set\_delta(delta);

23  sampler->get\_stepsize\_adaptation().set\_gamma(gamma);

24  sampler->get\_stepsize\_adaptation().set\_kappa(kappa);

25  sampler->get\_stepsize\_adaptation().set\_t0(t0);

26

27  sampler->engage\_adaptation();

28

29  try {

30  sampler->z().q = cont\_params;

31  sampler->init\_stepsize();

32  } catch (const std::exception& e) {

33  std::cout << "Exception initializing step size." << std::endl

34  << e.what() << std::endl;

35  return false;

36  }

37

38  return true;

39  }

40

41  template<class Sampler>

42  bool init\_adapt(stan::mcmc::base\_mcmc\* sampler,

43  stan::gm::categorical\_argument\* adapt,

44  const Eigen::VectorXd& cont\_params) {

45

46  double delta = dynamic\_cast<stan::gm::real\_argument\*>(adapt->arg("delta"))->value();

47  double gamma = dynamic\_cast<stan::gm::real\_argument\*>(adapt->arg("gamma"))->value();

48  double kappa = dynamic\_cast<stan::gm::real\_argument\*>(adapt->arg("kappa"))->value();

49  double t0 = dynamic\_cast<stan::gm::real\_argument\*>(adapt->arg("t0"))->value();

50

51  Sampler\* s = dynamic\_cast<Sampler\*>(sampler);

52

53  return init\_adapt<Sampler>(s, delta, gamma, kappa, t0, cont\_params);

54  }

55

56

57  }

58 }

59

60 #endif

base\_mcmc.hpp

categorical\_argument.hpp

stan::gm::singleton\_argument

**Definition:** singleton\_argument.hpp:44

stan::gm::categorical\_argument::arg

argument \* arg(const std::string name)

**Definition:** categorical\_argument.hpp:143

stan::gm::categorical\_argument

**Definition:** categorical\_argument.hpp:11

stan::common::init\_adapt

bool init\_adapt(Sampler \*sampler, const double delta, const double gamma, const double kappa, const double t0, const Eigen::VectorXd &cont\_params)

**Definition:** init\_adapt.hpp:13

Eigen.hpp

stan::math::e

double e()

Return the base of the natural logarithm.

**Definition:** constants.hpp:86

singleton\_argument.hpp

stan::agrad::log

fvar< T > log(const fvar< T > &x)

**Definition:** log.hpp:15

stan::mcmc::base\_mcmc

**Definition:** base\_mcmc.hpp:13


---

[ Stan Home Page ]

*© 2011–2014,
Stan Development Team.*
